# Hormonal Correlates of Exploratory and Play-Soliciting Behavior in Domestic Dogs

**DOI:** 10.3389/fpsyg.2018.01559

**Published:** 2018-09-10

**Authors:** Alejandra Rossi, Francisco J. Parada, Rosemary Stewart, Casey Barwell, Gregory Demas, Colin Allen

**Affiliations:** ^1^Laboratorio de Neurociencia Cognitiva y Social, Facultad de Psicología, Universidad Diego Portales, Santiago, Chile; ^2^Center for the Integrative Study of Animal Behavior, Indiana University, Bloomington, IN, United States; ^3^Cognitive Science Program, Indiana University, Bloomington, IN, United States; ^4^Program in Neuroscience, Indiana University, Bloomington, IN, United States; ^5^College of Veterinary Medicine, Ohio State University, Columbus, OH, United States; ^6^Department of History and Philosophy of Science, University of Pittsburgh, Pittsburgh, PA, United States

**Keywords:** exploration, play, oxytocin, cortisol, domestic dog

## Abstract

Exploration and play are considered to be crucial behaviors during mammalian development. Even though the relationship between glucocorticoids and exploratory behavior, stress, and anxiety is well described in the literature, very little is known about their role in play behavior in non-rodents. Likewise, the functional role of the “social hormone” oxytocin in exploration, play, stress, and anxiety is still unknown. The present work addresses this literature gap by studying plasma hormone profiles for cortisol (CORT) and oxytocin (OT) of domestic dogs exposed to a novel arena containing two unfamiliar trainers who did not interact with the dogs. We provide evidence suggesting a functional relationship between hormonal measures of cortisol and oxytocin and adaptive behavior (play-soliciting and exploration) in freely behaving domestic dogs. We have taken into account several possible factors in our analyses and interpretations, from the nature and quality of the measurements to demographic factors to statistical robustness. Our results indicate that reduced CORT levels are associated with increments of both play-soliciting behavior frequency and exploratory behavior duration. Furthermore, taken together, our data and our simulations suggest a relationship between OT and the enactment of play-soliciting behaviors by freely behaving domestic dogs that must be further investigated. Future studies should consider naturalistic structured and semi-structured experimental approaches linking behavior with (neuro) physiological measures, taking into account demographic factors such as age and relevant interphase factors such as the sex of the dog; and socio-historic factors such as the playfulness of the dog, history of interaction with young humans, among others, to take full account of interaction between humans and animals in comparative studies (Parada and Rossi, 2018).

## Introduction

Exploration and play are considered linked behavioral strategies for dealing with novelty, particularly during early mammalian development (Fiske and Maddi, 1961; Power, 2000; Burghardt, 2005). Their relevance to learning, predation strategies, and even tool use in species that use tools are long lasting (Rumbaugh et al., 1972; Hall and Bradshaw, 1998; Kramer and Burghardt, 1998). They have been conceptualized as an intertwined construct that shapes the way individuals face novelty during their life, and tending to occur when no other behavioral tendencies are active (Immelmann and Beer, 1989). Furthermore, they might be functionally similar, since they provide opportunities for adaptively shaping individuals’ knowledge and behaviors to the world.

In domestic dogs, both exploratory and play behavior tendencies may have facilitated the speciation process, since wild canids displaying more exploratory and playful behaviors (i.e., decreased flight and increased sociality) might have been taken as pets and socialized into human groups (Belyaev et al., 1985; Clutton-Brock, 1995; Driscoll et al., 2009; Driscoll and Macdonald, 2010; Miklósi and Topál, 2013; Range and Virányi, 2014). The fact that social play in dogs persists into adulthood contributes to their appeal as human companions (Bekoff, 1995). A developmental perspective on young animals’ exploratory responses to novelty suggests that not only do such responses determine survival but, over time, they lay the foundations for individual differentiation in how animals adapt to their environments (Parada and Rossi, 2018). Exploration, which involves the active investigation of the environment by an individual in the absence of pressing physiological needs (Immelmann and Beer, 1989), entails a particular interaction between the organism and its surrounding area, potentially shaping both of them. This probably makes exploration one of the driving forces in evolution (Greenberg and Mettke-Hofmann, 2001; Lefebvre et al., 2004). Exploration can be viewed as a type of information seeking about sources of food, mates or any unknown resource that might bridge the gap between an organism’s current state and states that are better adapted to current conditions. However, exploring novel environments can increase environmental risk factors, such as predation and aggression (Brown and Nemes, 2008). Exploration thus involves tradeoffs between benefits and risks in the course of encountering new situations and new potential play partners.

Play can be classified into either locomotor play, social play, individual play, or object play, although these are not necessarily mutually exclusive categories (Mehrkam et al., 2017). Social play, an apparently purposeless motor activity directed toward another agent and varied in both form and temporal sequencing (Bekoff and Byers, 1981, 1998), is built upon cooperation with the play partner, thus it is to be expected that pro-social mechanisms are important to sustaining it (Spinka et al., 2001; Bekoff, 2018). Social play is performed more frequently and for longer periods than either object or solitary play in many species (Burghardt, 2005). In common with other mammals, canids have a number of play-soliciting behaviors, such as approaching with an exaggerated, high-amplitude gait that is sometimes referred to as “loose” or “bouncy.” In addition, canids have evolved their own easily recognized social play-soliciting signals, such as play bows in which the shoulders are lowered below the level of the hips, and face pawing in which a dog lifts one of its front paws from the ground and directs it at the face of another dog, sometimes making contact (Bekoff, 1972, 1974).

In the domestic dog (*Canis lupus familiaris*), social play is very common, although unlike other canids the repertoire has expanded from conspecific play to dog-owner play, which is the more commonly seen form of social play. Dog–dog play and dog-owner play are possibly not homologous because they appear to be motivationally distinct (Rooney et al., 2000). Nevertheless, dogs direct many of the play-soliciting behaviors to humans just as easily as to other dogs. Practically all the studies on dog social play show similar results, generally indicating that social play in dogs is a marker of healthy development and positive affect, with long lasting effects on human-dog social cohesion (Horowitz and Hecht, 2016; Sommerville et al., 2017). Social play behavior has been described as an essential component of social development of animals, seemingly equipping animals with skills and strategies to deal with a variety of behaviors expressed in adulthood (Wang et al., 2012). Therefore, social play might be understood as a part of a “prosocial toolkit” that needs to be rehearsed and developed in order to facilitate the establishment of longer-term social ties (Vanderschuren et al., 1997; Vanderschuren, 2011).

Investigation of physiological mechanisms is an important element of integrated explanations in ethology (Tinbergen, 1963). Physiological measures associated with exploratory behavior, frequently related to stress and anxiety responses, have been thoroughly studied in many species (Greenberg, 1985; Koolhaas et al., 1997, 1999; Dingemanse and de Goede, 2004; Becker et al., 2007). Glucocorticoid response during exploration has been particularly well characterized in mammals, showing in general, that steroid hormone levels are negatively associated with exploratory behavior (e.g., Pellow et al., 1985; Carlstead et al., 1993; Conrad et al., 1997; Kunzl et al., 2003; Kazlauckas et al., 2005). Consequently, it has been shown that cortisol (CORT) is part of the stress response in mammals, which in turn makes it linked to reduced proclivity to interact with new objects or spaces. Notably, it has been shown that domesticated animals show lower glucocorticoid levels and more frequent exploratory behaviors relative to undomesticated individuals (Hemmer, 1990; Trut et al., 2004).

Hormonal correlates of social play have been investigated mainly in rodent species (Vanderschuren et al., 1997; Pellis and Pellis, 1998; Trezza et al., 2010; Taylor et al., 2012). There are very few studies exploring this link in other domesticated species (Sachs and Harris, 1978; Orgeur, 1995; Nunes et al., 1999). However, Horváth et al. (2008) showed that differences in the way humans interact with dogs in a playful interaction (affiliative vs. disciplinary) affect the cortisol levels of the dogs; an affiliative style decreased cortisol levels whereas a disciplinary one increased the hormone levels.

Strikingly, although social play is considered an important component of social behavior and has been carefully studied in canids (Bekoff, 1995; Bekoff and Allen, 1998; Rooney et al., 2001), the relationship between play behavior and both cortisol and the so-called “social hormone” oxytocin (OT) has only been recently explored. Recent evidence shows that salivary oxytocin in dogs is significantly increased after affiliative human–dog interaction (MacLean et al., 2017). This is relevant since oxytocin may promote socialization by its anxiolytic effects (Lancaster et al., 2017); for instance by promoting social play, especially in novel situations (for a review Kis et al., 2017). Similarly, recent evidence shows the effects of intranasal OT administration on dog behavior (Romero et al., 2014; Kis et al., 2015; Nagasawa et al., 2015; Oliva et al., 2015; Romero et al., 2015). These studies show a link between intranasal OT administration and affiliative behaviors directed by dogs toward their owners and toward other familiar dogs, suggesting that the administration of intranasal OT increases affiliative behaviors in dogs in a social context.

Relevantly, one study shows that intranasal OT administration increased the amount of play signals dogs gave to both familiar humans and conspecifics (Romero et al., 2015). Together, this evidence shows that the intranasal administration of OT triggered higher levels of affiliation, social orientation/approach, and gazing toward familiar individuals (Romero et al., 2014, 2015; Nagasawa et al., 2015). Collectively these results suggest that, similar to human-based studies, OT might help reveal the mechanisms of cooperation, and might also be essential to the behavioral displays that constitute the basis for the formation of social bonds.

The present study aims to explore the associations among exploratory and play-soliciting behaviors and plasma hormone measurements of cortisol and oxytocin in the domestic dog. To accomplish this, dogs were allowed to freely move about a novel arena for 10 min while being observed and video-recorded by two experimenters. Immediately following the behavioral trial, blood samples were collected and analyzed for cortisol (the predominant glucocorticoid in canids) and oxytocin. We hypothesized that (i) according to the literature showing that levels of cortisol modulate exploratory behavior in mammals, dogs’ exploratory behavior would be negatively correlated with cortisol concentrations and (ii) since peripheral oxytocin levels increase in both humans and dogs as a result of physical contact mostly in affiliative contexts (Odendaal and Meintjes, 2003; Handlin et al., 2011; Mitsui et al., 2011; Rehn et al., 2014), oxytocin levels would be positively correlated with a previously defined suite of play-soliciting behaviors in dogs (Bekoff, 1972, 1974).

## Materials and Methods

### Animals and Behavioral Sessions

Purebred or mixed breed Labrador retriever dogs (*n* = 14, mean age: 4.1 years, 10 male, 4 female, see **Table 1** for details) privately owned by local families served as study subjects. All subjects were naïve to the present study and to both the arena and the experimenters involved. This study was carried out in accordance with the approval of the Bloomington Institutional Animal Care and Use Committee (BIACUC, protocol 12-016). All owners signed a consent form prior to the session and they did not interact in any way with the experimenters or with their dogs after the drop-off. Testing was conducted at a local facility that provides canine training, daycare and veterinary services in Bloomington, IN, United States.

**Table 1:** Demographic data: Sex, weight (kilograms), age (years).

Demographic data			
Dog ID	Sex	Weight	Age
1	F	23.3	12
2	F	45.9	6
3	F	35.6	4
4	F	29.6	3
5	M	30.11	2
6	M	34.9	2.5
7	M	31.9	5
8	M	33.4	6
9	M	28.1	0.7
10	M	43.7	1
11	M	29.1	0.6
12	M	43.8	12
13	M	32.4	1
14	M	34.2	2

The sessions were carried out on separate days; thus, only one dog was tested on each day. Dogs were brought to the facility by their owners, who then left the facility. Each dog was placed alone in a grooming room kennel for between 10 and 15 min while the experimenters prepared the room. When the room was ready, they were fetched by one of the experimenters (experimenter 1 or E1) who walked them through the door. At the door, E1 took off the dogs’ walking collar allowing them to enter a clean, disinfected training room (17 × 15 m) clear of toys and other small objects. This room had concrete floors and walls, no windows but two doors, one at the front and the other one in the back of the room with small windows on top of them. Dogs could behave freely for 10 min. All sessions took place between 0830 and 0930 EST.

Two video cameras simultaneously recorded the dogs’ behavior during this time. Experimenter 1 (E1), who entered with the dog, stood then in the middle of the arena not moving from that location, but turning with the dog to face it without interacting with it during the entire session. E1 wore an ear-mounted digital camera (Looxcie 2). Experimenter 2 (E2) sat on a chair in a corner of the room and made no eye contact nor interacted in any manner with the dog. E2 handled a Sony HDR-CX160 video camera to capture the dogs’ behavior from different angles. Two active behavioral categories were coded and calculated for the exactly 10 min session: exploratory behavior (relative duration) and play-soliciting behavior (frequency), along with more passive behaviors such as sitting or lying down. These behaviors were treated as mutually exclusive. According to previous studies, exploratory behavior was operationally defined as locomotive behavior usually accompanied by sniffing and distal or close visual inspection in a relaxed manner (Prato-Previde et al., 2003; Rehn et al., 2013; Marshall-Pescini et al., 2017); play-soliciting behavior was coded using behavioral features adapted from the categories used by Bekoff (1972, 1974). We coded the behavior as play soliciting when it fell into any these categories: play bow (shoulders lowered beneath hips, forelimbs on, or near ground), exaggerated approach (approach with higher amplitude stepping or tail wagging than a normal approach), approach/withdrawal (dog approaches then abruptly turns and runs; typically used to solicit chasing behavior), paw intention (contact to person with paw off the ground), and leap-leap (two high-amplitude leaps in which the forelimbs are lifted off the ground, and hit the ground, simultaneously). These behaviors, and proximity were coded and quantified using the ELAN software (Lausberg and Sloetjes, 2009) by two independent coders who were trained to recognize these behaviors but blind to dogs’ hormone concentrations. The inter-rater correlations for exploratory and play-soliciting behaviors were 0.88 and 0.91, respectively (*p* < 0.001). Raw data were converted into frequencies and relative duration using custom in-house routines written in the MATLAB environment (The MathWorks, Inc., Natick, MA, United States).

### Blood Collection and Hormone Assays

At the end of the session, subjects were led to the veterinary clinic and blood was collected into chilled EDTA-treated tubes within 4 min to assess physiological status. Blood was drawn with a 22-gauge needle from the cephalic vein (1.5cc–3cc drawn). Half the sample was transferred to a second tube and immediately treated with aprotinin (500 KIU/ml) to inhibit protease activity. Samples were centrifuged (4°C, 1500 × *g*, 15 min) and plasma was stored in polypropylene tubes at −80°C until analysis. Cortisol was measured in untreated plasma using an enzyme immunoassay (EIA) kit (901-701; Enzo Life Sciences). Samples were diluted 1:4 and assayed in duplicate according to the manufacturer’s instructions. Serial dilution of pooled dog plasma yielded a displacement curve parallel to the cortisol standard curve (*r*^2^ = 0.98). Mean intra-assay variability was 3.7% and inter-assay variability was 4.6% (*n* = 3 plates). Aprotinin-treated plasma was assayed for oxytocin using a commercial EIA kit (900-153; Enzo Life Sciences) according to the manufacturer’s instructions. Samples were diluted 1:4 and assayed in duplicate on a single plate; three dogs (one female and two males, see **Table 1** for details) were removed from the analysis due to insufficient volume. Serial dilution of pooled dog plasma yielded a displacement curve parallel to the oxytocin standard curve (*r*^2^ = 0.98). Recovery of known amounts of oxytocin standard added to a pool of plasma extracts was 100.7 ± 11.7% (*y* = 1.04*x* + 1.1; *r*^2^ = 0.99). Mean intra-assay variability was 3.8%. Data summary is presented in **Table 2**.

**Table 2:** Cortisol and oxytocin levels (picograms per milliliter) and the frequency and duration for each behavior during 10 min dog spent with experimenters E1 and E1.

Data					
Dog ID	CORT (pg/mL)	OT (pg/mL)	Exploratory behavior duration (min)	Play-soliciting behavior (counts)	Sit/Stand/Lay < 2 m duration (min)
1	5855.56	NaN	3.24	1	6.89
2	7631.32	159.9	1.64	0	8.3
3	1462.48	392.1	6.25	23	1.71
4	1267.63	161.1	5.14	15	2.69
5	4148.59	NaN	4.74	28	0.26
6	1304.09	162.1	5.86	15	0.17
7	2340.07	50.9	3.82	1	5.92
8	1099.43	158.5	8.85	7	0
9	2768.5	NaN	9.91	2	0
10	8518.74	114.9	0.95	0	8.7
11	3286.77	287.6	9.39	11	0
12	2706.47	79	4.8	4	5.09
13	1106.17	225.7	9.39	11	0
14	7538.05	162.2	5.15	7	4.11

### Statistical Analyses

To statistically test our main hypotheses – relationship between both exploratory and play-soliciting behaviors and hormone concentrations – we implemented hierarchical multiple regression in order to further explore the influence between physiological (OT, CORT) and the most relevant demographic factors (age, sex) over exploratory and play-soliciting behavior. In view of both physiological measures, we built the first block of predictors using the *forced entry* method. In order to build the whole model, the second block included both physiological and demographic predictor variables (OT, CORT, Age, Sex). Preliminary analyses were performed ensuring no violation of multilinear regression assumptions (Durbin–Watson = 2.345, 1.838; Standardized residuals <1.943). The weight predictor was left out of the model due to high multicollinearity (VIF > 5). Furthermore, collinearity analyses showed that our four predictors were within analysis range, possible multicollinearity was discarded when using four predictors and no other factors were removed (Tolerance >0.561; VIF <1.781).

**FIGURE 1:**
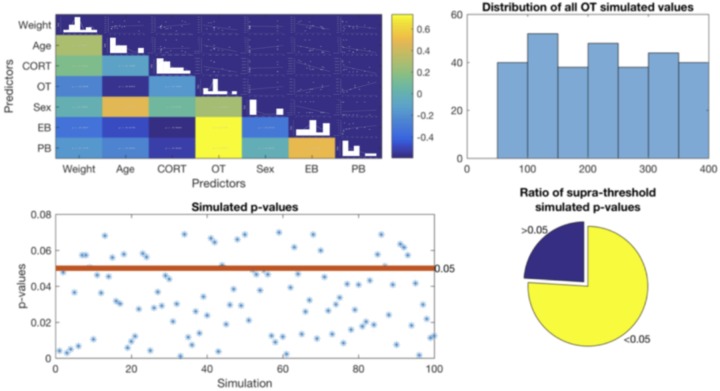
Top Left – Correlation matrix between all variables. Top right – distribution of all OT simulated values showing no tendency. Bottom left – all *p*-values obtained from the simulated data. Red line indicates the considered statistical threshold, less than 30% of these *p*-values were above threshold (bottom right).

Considering the number of study subjects and the missing data points in OT measurement, we implemented a complementary analysis. Given the missing data points for OT measurements from three study subjects, we created a simulated dataset using the non-parametric permutation framework implemented in the MATLAB environment. Thus, 100 new datasets were constructed without missing values. The simulated 300 values were drawn from a distribution with similar parameters as the original data (range = 50.9+/−2 std to 392.1+/−2 std; mean = 177.6; median = 161.1, **Figure 1**). The 100 complete simulated datasets were also analyzed using hierarchical multiple regression procedure described above.

All statistical analyses were performed using JASP software version 0.8.5.1 (JASP Team, 2016).

## Results

### Main Analysis: Hierarchical Multiple Regression

Physiological predictors were entered in the first step of hierarchical multiple regression (**Table 3**, model 0: OT, CORT), explaining 59% of exploratory behavior variance [*R*^2^ = 0.586; $R_{\text{adj}}^{2}$ = 0.482, *F*(2,8) = 5.653, *p* = 0.029] and 80% of play-soliciting behavior variance [*R*^2^ = 0.801; $R_{\text{adj}}^{2}$ = 0.752, *F*(2,8) = 16.126, *p* = 0.002]. Both models including only physiological predictors were statistically significant. Observation of Beta (β) coefficients indicates that CORT made a statistically significant contribution for both exploratory behavior (β = −0.601, *p* = 0.034) and play-soliciting behavior (β = −0.458, *p* = 0.023), while OT contributed significantly only to play-soliciting behavior (β = 0.665, *p* = 0.003). Demographic variables were included in order to build the final model in step 2 (**Table 3**, model 1: Sex, Age, OT, CORT). The model as a whole explained 80% of exploratory behavior variance [*R*^2^ = 0.799; $R_{\text{adj}}^{2}$ = 0.665, *F*(4,6) = 5.954, *p* = 0.028] and 83% of play-soliciting behavior variance [*R*^2^ = 0.829; $R_{\text{adj}}^{2}$ = 0.714, *F*(4,6) = 7.256, *p* = 0.018]. Including demographic variables significantly improved both models. However, the additional 21% of exploratory behavior variance was not a statistically significant change [*R*^2^ change = 0.213, *F*(2,6) = 3.178, *p* = 0.115]. Similarly, adding demographic variables only increased 3% of play-soliciting behavior explained variance, not yielding statistically significant results [*R*^2^ change = 0.027, *F*(2,6) = 0.640, *p* = 0.640].

**Table 3:** Hierarchical multiple regression models summary.

Model	*R*	*R*^2^	Adjusted *R*^2^	RMSE	*R*^2^ change	*F* change	df1	df2	*p*	Durbin–Watson
**Exploratory behavior model summary**										
0	0.765	0.586	0.482	2.052	0.586	5.653	2	8	0.029	
1	0.894	0.799	0.665	1.651	0.213	3.178	2	6	0.115	2.345

*Null model 0 includes OT (pg/mL), CORT (pg/mL).*										

**Play-soliciting behavior model summary**										
0	0.895	0.801	0.752	3.638	0.801	16.126	2	8	0.002	
1	0.910	0.829	0.714	3.900	0.027	0.480	2	6	0.640	1.838

*Null model 0 includes CORT (pg/mL), OT (pg/mL).*

**Table 4:** Hierarchical multiple regression model ANOVA.

Model		Sum of squares	df	Mean square	*F*	*p*
**Exploratory behavior model ANOVA**						
0	Regression	47.61	2	23.805	5.653	0.029
	Residual	33.69	8	4.211		
	Total	81.30	10			
1	Regression	64.94	4	16.234	5.954	0.028
	Residual	16.36	6	2.726		
	Total	81.30	10			

*Null model 0 includes OT (pg/mL), CORT (pg/mL).*						

**Play-soliciting behavior model ANOVA**						
0	Regression	426.85	2	213.42	16.126	0.002
	Residual	105.88	8	13.23		
	Total	532.73	10			
1	Regression	441.46	4	110.37	7.256	0.018
	Residual	91.26	6	15.21		
	Total	532.73	10			

*Null model 0 includes CORT (pg/mL), OT (pg/mL).*

Therefore, in the final model, β coefficients indicate that CORT made a significant unique contribution to both exploratory (β = −0.557, *p* = 0.032) and play-soliciting (β = −0.514, *p* = 0.032) behaviors, while OT might have a contribution to play-soliciting behavior, which cannot be untangled with our current dataset (β = 0.525, *p* = 0.057, see “Discussion” section). All other β coefficients were well above statistical threshold (*p* > 0.05). See **Tables 3**, **4**, and **Supplementary Table 1** for model ANOVA results and model coefficients.

### Supplementary Analysis: Hierarchical Multiple Regression With Simulated Data

As expected, the first level of our simulation results replicated the findings of the main analysis hierarchical model. That is, CORT was negatively correlated with both exploratory and play-soliciting behaviors, while OT was positively correlated only with play-soliciting behavior. Likewise, the simulation further shows that although adding demographic predictors increases the overall model performance, the *R*^2^ change is not statistically significant. Thus, the second level of our simulation partially replicates the main analysis final model. Accordingly, CORT robustly shows a negative correlation with both exploratory and play-soliciting behaviors. However, the simulation is not congruent with our findings regarding OT. Our simulated results show that fewer than 30% of simulations replicate our OT hierarchical multiple regression analysis (β > 0.544 ± 0.065, *p* < 0.074 ± 0.018). This indicates no statistically significant contribution of OT to play-soliciting behavior. In contrast, the other >70% of simulations suggest a significant OT contribution to play-soliciting behavior (β > 0.661 ± 0.086, *p* < 0.027 ± 0.004), **Figure 1**. We will discuss this discrepancy in the following section.

## Discussion

The present study provides evidence for a link between behavior of dogs in a novel setting and physiological measures taken immediately after. Thus presenting new evidence about the relationship among cortisol, oxytocin and exploratory and play-soliciting behaviors in freely behaving domestic animals. Along with confirming an expected link between CORT and exploratory and play-soliciting behavior, this is the first study, to our knowledge, presenting data and a simulation associating oxytocin with play-soliciting behavior.

Our main analysis, a two-level hierarchical multiple regression, suggested that reduced CORT predicts an increment of both play-soliciting behavior frequency and exploratory behavior duration. These results are in line with previous studies (e.g., Lupien and McEwen, 1997; Kalivas and Nakamura, 1999), and its robustness is seen on both levels of the main analysis as well as both levels of the simulation. Therefore, our overall results confirm that decreased CORT levels predict both exploratory behavior duration and play-soliciting behavior frequency.

Furthermore, the first level of our analysis suggests that increased levels of OT might be relevant for frequency increments of play-soliciting behavior. However, while demographic factors failed to make statistically significant contributions, when added to the model they suggest that the role of OT in play-soliciting behavior is not conclusive (with a *p*-value of 0.057). Moreover, our supplementary analysis outcome is divided regarding OT’s contribution to play-soliciting behavior, as less than 30% of simulations follow the non-conclusive result of the main analysis. In other words, adding demographic factors to the hierarchical multiple regression models decreases the statistical compatibility of the observed OT relationship with increasing play-soliciting behavior frequency. Although there are many reasons to explain the slight OT *p*-value increment (a 0.047 difference between models) we have enough evidence to support the discussion of three factors: (i) low subject sample, (ii) sex of subjects, and (iii) missing values for OT readings, and we will proceed to address them.

Regarding the low subject sample, we would like to point at the fact that these kind of studies are expensive and complicated to implement: (i) it is difficult to find domestic dogs whose owners are willing to allow drawing blood for data acquisition, (ii) locating facilities willing to host such research is onerous, and (iii) acquiring necessary resources including financial support is always burdensome.

Regarding imbalanced sex ratio, it must be said that it is a function of low subject sample attributable to the challenge of recruiting willing owners. Even though sex, as a relevant predictor, was not consistent enough to have a significant impact on our results, we interpret the result from the full exploratory behavior model (β = −0.5, *p* = 0.059) as providing evidence that future studies should make all possible efforts to equalize sex ratio in the sample.

Finally, regarding the missing OT values, the results in our second supplementary analysis using 100 simulated datasets suggest that the negative relationship between CORT levels and both exploratory behavior duration and play-soliciting behavior frequency is robust, since it was consistently present in both models (with and without demographic predictors). The simulation further confirms that although adding demographic predictors increase the overall model performance for both behaviors, the R^2^ changes are not large enough to become relevant. Furthermore, the positive relationship between OT and play-soliciting behavior remained intact in the first step of all hierarchical models using simulated data (no demographic data included). However, when demographic predictors were added in the second step, more than 70% of our simulated hierarchical models suggested that increased OT levels made a unique and significant contribution to incrementing play-soliciting behavior frequency (**Figure 1**). The discrepancy between the original hierarchical multiple regression analysis and the simulation suggests that the missing OT measurement values might be a more important factor to account for the observed 0.047 *p*-value increment when adding demographic factors into the hierarchical model.

Taken together, our analyses not only support but also expand our first hypothesis, suggesting that reduced CORT levels are linked to both the duration of exploratory behaviors and the frequency of play-soliciting behaviors in freely behaving domestic dogs. We interpret this relationship as a physiological signature of an *openness* to explore for longer times and consequently, to engage in more frequent interactions, such as play-soliciting behaviors. Furthermore, our results suggest a possible relationship between OT and the enactment of play-soliciting behaviors by freely behaving domestic dogs that must be further investigated.

### Directions for Future Work

Previous studies have shown anxiolytic-like, stress-reducing effects of oxytocin in mammals (Uvnas-Moberg et al., 1994; Windle et al., 1997). Thus, it is possible that reduced fear relates to approach responses and *perhaps* exploratory behavior in dogs, evidenced by reduced CORT and possible increased OT.

**FIGURE 2:**
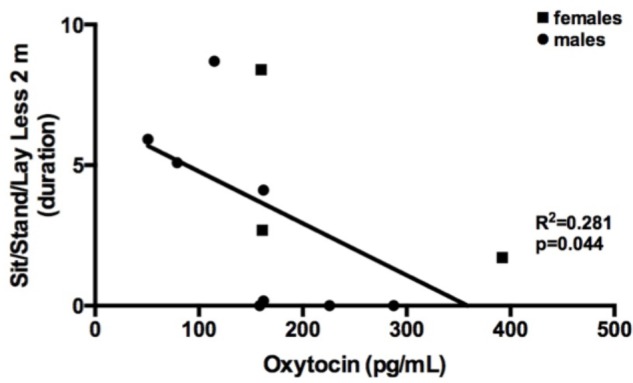
Correlation between Standing, seating, laying less than 2 m away from the experimenters (relative duration) and plasma oxytocin (pg/mL).

The possibly reciprocal and/or regulatory relationship between OT and CORT is still an open question. However, the origin of the physiological relationship between CORT, OT, and exploratory and play-soliciting behaviors might lie in the domesticated nature of *Canis lupus familiaris* (Hemmer, 1990; Trut et al., 2004). In canids, research has shown that both dog and owner oxytocin levels increase after positive social interactions (Miller et al., 2009; Nagasawa et al., 2009; Handlin et al., 2011). In our study the experimenters did not directly interact with dogs, remaining neutral during the session. Furthermore, dogs displaying physical proximity between 0 and 2 m to the experimenters showed overall lower levels of oxytocin concentrations; the relationship between dogs’ oxytocin levels and play-soliciting behavior is not explained by physical proximity between the dog and the experimenters (**Figure 2**).

The role of hormones in processes such as exploratory and play-soliciting behavior that enable dogs to deal with novel anthropogenic environments and which may have contributed to the domestication of dogs from wolves (Belyaev et al., 1985; Trut et al., 2004), should be further explored. We believe that our present study contributes with more evidence to the current line of research of causal mechanisms by manipulating these hormones in domestic dogs using methods already considered acceptable for research on human subjects such as hormonal administration (e.g., Romero et al., 2015; Kis et al., 2017; Persson et al., 2017; Temesi et al., 2017 for a critical review).

## Conclusion

This article provides some evidence of a functional relationship between hormonal measures of CORT and OT and adaptive behavior (play-soliciting and exploration) in freely behaving domestic dogs. We have taken into account several possible factors in our analyses and interpretations, from the nature and quality of the measurements to demographic factors to statistical robustness. Future studies should consider naturalistic structured and semi-structured experimental approaches linking behavior with (neuro) physiological measures, taking into account demographic factors such as age and relevant interphase factors such as the sex of the dog; and socio-historic factors such as the playfulness of the dog, history of interaction with young humans, among others, to take full account of interaction between humans and animals in comparative studies (Parada and Rossi, 2018).

## Author Contributions

AR and CA conceptualized the research. AR, CA, GD, and CB designed the experiments. AR and CB collected behavioral and physiological data. AR and RS analyzed the physiological data. FP and AR implemented the statistical analyses. AR, FP, CA, and GD wrote and edited the article.

## Conflict of Interest Statement

The authors declare that the research was conducted in the absence of any commercial or financial relationships that could be construed as a potential conflict of interest.
