# Supplementary material for: Hormonal Correlates of Exploratory and Play-Soliciting Behavior in Domestic Dogs
Source: Front Psychol. 2018 Sep 10;9:1559. doi: 10.3389/fpsyg.2018.01559 (PMC6139352; doi:10.3389/fpsyg.2018.01559)
Supplement: Supplementary file 1 [file Table_1.DOCX]

Supplementary Table 1. All hierarchical multiple regression model coefficients

| **Exploratory Behavior Model Coefficients** | | | | | | | | | | | | | | | | | | | | | | | |  |
| --- | --- | --- | --- | --- | --- | --- | --- | --- | --- | --- | --- | --- | --- | --- | --- | --- | --- | --- | --- | --- | --- | --- | --- | --- |
|  | | | | | | | | | | | | | | | | | **Collinearity Statistics** | | | | | | |  |
| **Model** | |  | | **Unstandardized** | | **Standard Error** | | **Standardized** | | **t** | | **p** | | **2.5%** | | **97.5%** | **Tolerance** | | | | **VIF** | | |  |
| 0 |  | (Intercept) |  | 5.749 |  | 1.742 |  |  |  | 3.300 |  | 0.011 |  | 1.731 |  | 9.766 |  |  |  | | | |  |  |
|  |  | OT(pg/mL) |  | 0.010 |  | 0.007 |  | 0.350 |  | 1.490 |  | 0.175 |  | -0.006 |  | 0.027 |  | 0.941 |  | | | | 1.063 |  |
|  |  | CORT(pg/mL) |  | -5.841e -4 |  | 2.281e -4 |  | **-0.601** |  | -2.561 |  | **0.034** |  | -0.001 |  | -5.811e -5 |  | 0.941 |  | | | | 1.063 |  |
| 1 |  | (Intercept) |  | 5.426 |  | 2.073 |  |  |  | 2.617 |  | 0.040 |  | 0.352 |  | 10.499 |  |  |  | | | |  |  |
|  |  | Sex |  | -3.053 |  | 1.299 |  | **-0.500** |  | -2.350 |  | **0.059** |  | -6.232 |  | 0.126 |  | 0.740 |  | | | | 1.351 |  |
|  |  | Age(years) |  | -0.018 |  | 0.188 |  | -0.021 |  | -0.098 |  | 0.925 |  | -0.479 |  | 0.442 |  | 0.701 |  | | | | 1.426 |  |
|  |  | OT(pg/mL) |  | 0.016 |  | 0.007 |  | 0.553 |  | 2.264 |  | 0.064 |  | -0.001 |  | 0.034 |  | 0.561 |  | | | | 1.781 |  |
|  |  | CORT(pg/mL) |  | -5.414e -4 |  | 1.942e -4 |  | **-0.557** |  | -2.788 |  | **0.032** |  | -0.001 |  | -6.625e -5 |  | | 0.840 |  | | 1.190 |  |  |
|  | | | | | | | | | | | | | | | | | | | | | | | |  |

| **Play-soliciting Behavior Model Coefficients** | | | | | | | | | | | | | | | | | | | | | |
| --- | --- | --- | --- | --- | --- | --- | --- | --- | --- | --- | --- | --- | --- | --- | --- | --- | --- | --- | --- | --- | --- |
|  | | | | | | | | | | | | | | | | | | **Collinearity Statistics** | | | |
| **Model** | |  | | **Unstandardized** | | **Standard Error** | | **Standardized** | | **t** | | **p** | | **2.5%** | | **97.5%** | | **Tolerance** | | **VIF** | |
| 0 |  | (Intercept) |  | 3.496 |  | 3.089 |  |  |  | 1.132 |  | 0.290 |  | -3.627 |  | 10.618 |  |  |  |  |  |
|  |  | CORT(pg/mL) |  | -0.001 |  | 4.044e -4 |  | **-0.458** |  | -2.820 |  | **0.023** |  | -0.002 |  | -2.077e -4 |  | 0.941 |  | 1.063 |  |
|  |  | OT(pg/mL) |  | 0.051 |  | 0.012 |  | **0.665** |  | 4.094 |  | **0.003** |  | 0.022 |  | 0.079 |  | 0.941 |  | 1.063 |  |
| 1 |  | (Intercept) |  | 6.542 |  | 4.897 |  |  |  | 1.336 |  | 0.230 |  | -5.441 |  | 18.524 |  |  |  |  |  |
|  |  | Sex |  | 2.511 |  | 3.069 |  | 0.161 |  | 0.818 |  | 0.444 |  | -4.998 |  | 10.020 |  | 0.740 |  | 1.351 |  |
|  |  | Age(years) |  | -0.345 |  | 0.445 |  | -0.157 |  | -0.777 |  | 0.467 |  | -1.433 |  | 0.742 |  | 0.701 |  | 1.426 |  |
|  |  | CORT(pg/mL) |  | -0.001 |  | 4.587e -4 |  | **-0.514** |  | -2.787 |  | **0.032** |  | -0.002 |  | -1.560e -4 |  | 0.840 |  | 1.190 |  |
|  |  | OT(pg/mL) |  | 0.040 |  | 0.017 |  | **0.525** |  | 2.329 |  | **0.057** |  | -0.002 |  | 0.082 |  | 0.561 |  | 1.781 |  |
|  | | | | | | | | | | | | | | | | | | | | | |
